# Characteristics of a New X-Ray Imaging System for Interventional Procedures: Improved Image Quality and Reduced Radiation Dose

**DOI:** 10.1007/s00270-017-1821-z

**Published:** 2017-10-31

**Authors:** Ruediger E. Schernthaner, Reham R. Haroun, Sonny Nguyen, Rafael Duran, Jae Ho Sohn, Sonia Sahu, Julius Chapiro, Yan Zhao, Alessandro Radaelli, Imramsjah M. van der Bom, Maria Mauti, Kelvin Hong, Jean-François H. Geschwind, MingDe Lin

**Affiliations:** 1 Section of Cardiovascular and Interventional Radiology, Department of Biomedical Imaging and Image-guided Therapy, Medical University of Vienna, Währinger Gürtel 18-20, 1090 Vienna, Austria; 2 Department of Radiology and Biomedical Imaging, Yale University School of Medicine, 330 Cedar Street, TE 2-230, New Haven, CT 06520 USA; 3 Image-Guided Therapy Systems, Philips Healthcare, Best, The Netherlands; 4 Russell H. Morgan Department of Radiology and Radiological Science, Division of Vascular and Interventional Radiology, The Johns Hopkins Hospital, Baltimore, MD 21287 USA; 5 PreScience Labs, Westport, CT 06880 USA; 6 U/S Imaging and Interventions, Philips Research North America, Cambridge, MA USA

**Keywords:** Interventional radiology, Radiation dosage, Image quality enhancement

## Abstract

**Purpose:**

To compare image quality and radiation exposure between a new angiographic imaging system and the preceding generation system during uterine artery embolization (UAE).

**Materials and Methods:**

In this retrospective, IRB-approved two-arm study, 54 patients with symptomatic uterine fibroids were treated with UAE on two different angiographic imaging systems. The new system includes optimized acquisition parameters and real-time image processing algorithms. Air kerma (AK), dose area product (DAP) and acquisition time for digital fluoroscopy (DF) and digital subtraction angiography (DSA) were recorded. Body mass index was noted as well. DF image quality was assessed objectively by image noise measurements. DSA image quality was rated by two blinded, independent readers on a four-rank scale. Statistical differences were assessed with unpaired *t* tests and Wilcoxon rank-sum tests.

**Results:**

There was no significant difference between the patients treated on the new (*n* = 36) and the old system (*n* = 18) regarding age (*p* = 0.10), BMI (*p* = 0.18), DF time (*p* = 0.35) and DSA time (*p* = 0.17). The new system significantly reduced the cumulative AK and DAP by 64 and 72%, respectively (median 0.58 Gy and 145.9 Gy*cm^2^ vs. 1.62 Gy and 526.8 Gy*cm^2^, *p* < 0.01 for both). Specifically, DAP for DF and DSA decreased by 59% (75.3 vs. 181.9 Gy*cm^2^, *p* < 0.01) and 78% (67.6 vs. 312.2 Gy*cm^2^, *p* < 0.01), respectively. The new system achieved a significant decrease in DF image noise (*p* < 0.01) and a significantly better DSA image quality (*p* < 0.01).

**Conclusions:**

The new angiographic imaging system significantly improved image quality and reduced radiation exposure during UAE procedures.

**Electronic supplementary material:**

The online version of this article (doi:10.1007/s00270-017-1821-z) contains supplementary material, which is available to authorized users.

## Introduction

The continuous development of new techniques and indications in interventional radiology [1] has led to a steady increase in overall procedures since the 1950s [2]. Together with diagnostic radiology and nuclear medicine, medical imaging is the main contributor for man-made radiation exposure and accounted for approximately one-eighth of the worldwide exposure from man-made and natural radiation sources in 2004 [3]. Thus, the awareness and efforts for a better balance between radiation exposure and diagnostic image quality increased over the years—namely the “as low as reasonably achievable” principle (ALARA) [4]. This is especially important for image-guided procedures, where both patients and clinical staff are exposed to high doses of ionizing radiation [5].

A new X-ray imaging system (AlluraClarity; Philips Healthcare, Best, The Netherlands) was recently released that incorporates additional filtering and optimized acquisition protocols to lower the radiation dose at the cost of decreased image quality, and a workstation with real-time image processing algorithms to improve the image quality in order to maintain an adequate procedural performance. Several recently published studies confirmed that the new system resulted in a significant radiation exposure reduction during procedures in the fields of neuroradiology [6, 7], interventional oncology [8, 9] and interventional cardiology [10, 11]. However, the main objective of these studies was to show reduction in radiation exposure with equivalent image quality and procedural performance. Because this new imaging system includes several real-time image processing algorithms to improve image quality during digital subtraction angiography (DSA), we sought to investigate not only the dose-saving effect, but also the capabilities that yield superior image quality compared to the preceding generation imaging system. This applies in particular to procedures sensitive to motion artifacts, such as small bowel movement during uterine artery embolization (UAE).

Thus, the purpose of our study was to compare image quality and radiation exposure between this new angiographic imaging system and the preceding generation system during UAE.

## Materials and Methods

### Study Cohort

This retrospective, single-institution, two-arm study was approved by the institutional review board. All patients with uterine fibroids that were treated using uterine artery embolization (UAE) at our institution consecutively between May 2014 and April 2015 were retrospectively included in the study.

### MR Imaging Technique

All patients underwent baseline MR imaging for treatment planning [12] within 5 months before UAE (mean 71 days, range 9–131), using our institutional protocol, as previously described [13]. For each woman, the sagittal abdominal diameter was measured at the level of uterus on sagittal T1-weighted MR images.

### Angiographic Imaging Systems

The procedures were performed in two different angiographic suites: one being equipped with a recently released imaging system (AlluraClarity FD 20, Philips Healthcare, Best, The Netherlands; study group) and the other one with the preceding generation imaging system of the same vendor (AlluraXper FD 20; control group). Both imaging systems share a dynamic 14-bit flat panel digital detector with an image matrix of 2480 × 1920 pixels, a pixel pitch of 154 × 154 µm and a maximum field of view of 30 × 38 cm. Automatic tube current modulation was enabled on both systems. However, as previously mentioned, the new system used additional filtering (0.1-mm copper and 1-mm aluminum filters) and optimized acquisition protocols (a decreased tube voltage of 75 vs. 78 kVp and a smaller focal spot size of 0.4 vs. 0.7 mm) to lower the radiation exposure, and real-time image processing algorithms to compensate for the loss in image quality due to lower radiation flux (spatial noise reduction for digital fluoroscopy (DF) and spatial noise reduction, temporal averaging and automatic pixel shift for DSA [6]).

### Embolization Protocol

An experienced interventional radiologist (K.H., 10 years of experience in abdominal interventions) performed all embolization procedures. A consistent approach according to our standard institutional protocol was used for all patients [14].

### Radiation Exposure Measurements and Calculations

The new imaging system (used for the study group) supported Digital Imaging and Communications in Medicine (DICOM) Radiation Dose Structured Reports (RDSR). RDSR contains detailed log information of every X-ray event, including radiation time, air kerma (AK), dose area product (DAP) and number of images acquired. A dedicated workstation was set up with DoseUtility (PixelMed Publishing, Bangor, PA) to receive, store and evaluate the RDSRs of the study group patients.

The old imaging system (used for the control group) did not support RDSR; thus, the examination reports generated by the system were used. These examination reports contained the AK and the DAP of the entire procedure as well as the cumulative DAP for DF and DSA runs, respectively. However, the cumulative radiation time was only provided for DF runs, whereas for DSA runs, the number of acquired images was provided. Thus, the DSA radiation time had to be calculated using the number of images acquired during each run and knowing the frame rate used. For example, 15 frames at a frame rate of three frames per second correspond to a radiation time of 5 s. To prove that all these calculations were correct, examination reports of five patients undergoing UAE on the new system were also collected, and the calculated values were found to be the same as the values recorded using RDSR.

To compensate for the differences in procedure complexity and thus in radiation time between the patients, the recorded DAP values were normalized by the corresponding radiation times for both DF and DSA runs. For example, the normalized DAP for 1 s of DSA was calculated as

$${\text{DSA}}\;{\text{Cumulative}}\;{\text{DAP}}\; ( {\text{Gy}}*{\text{cm}}^{2} ) / {\text{DSA}}\;{\text{radiation}}\;{\text{time}}\;({\text{s}}).$$

### DF Image Quality Assessment

Objective DF image quality assessment was performed in a blinded fashion on an Osirix workstation (Pixmeo, Bernex, Switzerland) by an interventional radiologist (R.E.S.) with more than 5 years of clinical experience in UAE and corresponding imaging, who did not participate in the UAE procedures. A circular region of interest (ROI) with an area of 3 cm^2^ was placed on the iliac bone, avoiding gas-filled intestines, and the mean signal intensity and the standard deviation of pixels within the ROI were recorded. A signal-to-noise ratio (SNR) was calculated using the formula

$${\text{SNR}} = {\text{mean/standard}}\;{\text{deviation}}.$$

In addition, the signal intensity of the guidance wire was assessed by placing an elliptic ROI with an area of 3 cm^2^ on the wire and recording the minimum signal intensity within the ROI, which corresponds to the wire. A contrast ratio (CR) was calculated with the formula

$${\text{CR}} = {\text{mean/guidance}}\;{\text{wire}}.$$

### DSA Image Quality Assessment

Qualitative DSA image analysis was performed by two interventional radiologists (R.E.S. and R.D.) each with more than 5 years of clinical experience in UAE and corresponding imaging, who did not participate in the UAE procedures. The DSA images of both uterine arteries of all women were presented in a blinded and randomized fashion on an Osirix workstation. The window/level settings used were maintained to be the default settings in Osirix. Both readers determined independently of each other in separate reading sessions the visibility of the small feeding arteries of the uterine fibroids (parameter 1) as well as the absence of artifacts related to breathing (parameter 2) and small bowel movement (parameter 3) on a binary scale (yes = 1, no = 0). These three image quality parameters were summarized in a four-scale scoring system, where a score of 3 was considered best and a score of 0 was considered worst.

### Statistical Analysis

All statistical computations were performed in SPSS Statistics 23 (IBM Corp., Armonk, NY). A *p* value < 0.05 was considered statistically significant. Descriptive statistics were performed to summarize the data. The distribution of scale variables was assessed with the Shapiro–Wilk test. For scale variables with normal distribution, mean, standard deviation and range were used and an unpaired *t* test was performed. For scale variables with non-Gaussian distribution, median, interquartile range and range were used and a Wilcoxon rank-sum test was performed. For ordinal variables, median, count and percentage were used and a Wilcoxon rank-sum test was performed. For the assessment of interobserver agreement, Kendall’s tau coefficient was calculated.

## Results

### Patient Demographics and Radiation Time

Between May 2014 and April 2015, 54 consecutive women with uterine fibroids underwent baseline MRI and were treated using UAE at our institution. A total of 36 (66%) and 18 (33%) UAE procedures were performed on AlluraClarity FD 20 (study group) and on AlluraXper FD 20 (control group), respectively. There was no significant difference between the study and the control groups regarding baseline patient characteristics and radiation times, as shown in Table 1.

**Table 1:** Baseline characteristics and radiation time of all patients, the study and the control groups

Characteristic	All patients (*n* = 54)	Study group (*n* = 36)	Control group (*n* = 18)	*p* value
Age	45.4 ± 5.3 (30–58)	44.5 ± 5.7 (30–58)	47.1 ± 4.1 (37–53)	0.10
Body mass index	33.4 ± 7.7 (21.7–55.3)	32.2 ± 8.3 (21.7–55.3)	35.2 ± 6.4 (27.4–48.0)	0.18
Sagittal abdominal diameter at the level of the uterus (mm)	246 ± 35 (171–357)	244 ± 38 (171–357)	252 ± 29 (204–306)	0.41
DF time (min)*	24.6; 10.0 (14.2–43.7)	24.1; 9.8 (14.2–43.7)	26.6; 9.7 (15.5–42.5)	0.35
DSA time (s)*	63.6; 29.6 (37.8–115.5)	64.9; 29.7 (42.0–115.5)	58.6; 36.4 (37.8–109.0)	0.17

Except where indicated, data represented as mean ± standard deviation (range)

* Data represented as median and interquartile range (range)

### Radiation Exposure

**Fig. 1:**
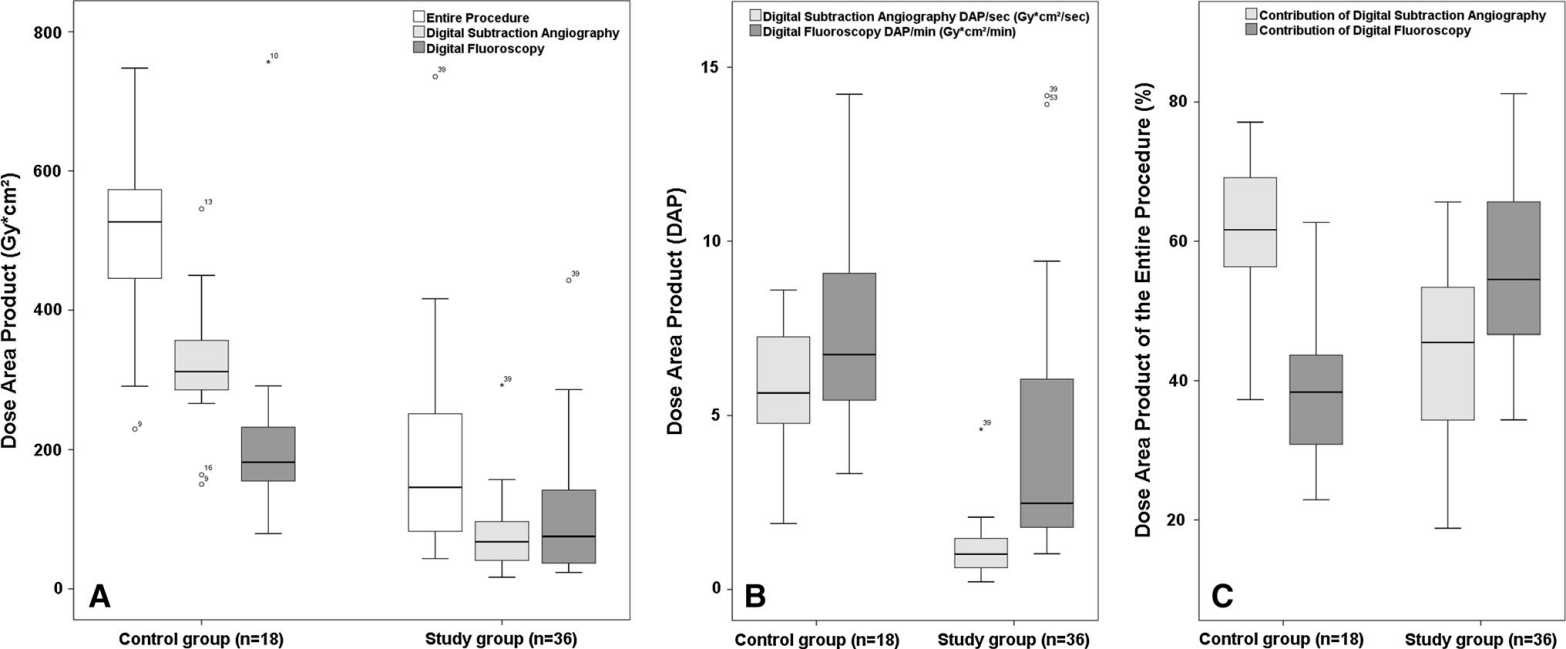
Box plots showing the radiation exposure of the control and study groups during the entire procedure, during all digital subtraction angiography (DSA) and all digital fluoroscopy (DF) runs (**A**), during 1 min of DF and 1 s of DSA (**B**) as well as the contribution of DF and DSA to the cumulative radiation exposure during the entire procedure (**C**). Each plot shows the interquartile range (box), 5th and 95th percentiles (outermost bars) and the median (thick horizontal line) of the exposure distribution in each system

The total DAP and AK of the entire procedure in the study group were 72% (145.9 vs. 526.8 Gy*cm^2^) and 64% (0.58–16.2 Gy) lower compared to those values in the control group, respectively. The new system yielded a DF and DSA DAP that was 59% (75.3 vs. 181.9 Gy*cm^2^) and 78% (67.6 vs. 312.2 Gy*cm^2^) lower compared to the old system, respectively (Fig. 1A). After normalizing DF and DSA by the radiation time, the DAP for DF and DSA decreased by 63% (from 6.75 to 2.48 Gy*cm^2^/min) and 82% (from 5.65 to 1.02 Gy*cm^2^/s), respectively (Fig. 1B). All these differences were statistically significant (*p* < 0.01).

In the control group, DF and DSA accounted for 38 ± 9 and 62 ± 9% of the cumulative DAP of the entire procedure, respectively. In the study group, the contribution of DF increased to 56 ± 12%, whereas the contribution of DSA to the cumulative DAP of the entire procedure decreased to 44 ± 12% (Fig. 1C). This composition of overall DAP was significantly different between the two patient groups (*p* < 0.01).

A detailed description of DAP and AK for the study and the control groups by means of median, interquartile range, minimum and maximum is provided in Table 2, including the values normalized by radiation time.

**Table 2:** Radiation exposure for the study and the control groups

	Study group	Control group	Reduction (%)	*p* value
DAP of the entire procedure (Gy*cm^2^)	145.9; 174.8 (43.4–735.6)	526.8; 128.7 (229.6–1206.8)	72	< 0.01
AK of the entire procedure (Gy)	0.58; 0.73 (0.20–2.50)	1.62; 0.53 (0.67–3.88)	64	< 0.01
DF DAP (Gy*cm^2^)	75.3; 108.1 (23.5–442.7)	181.9; 87.2 (79.3–756.8)	59	< 0.01
DF DAP/min (Gy*cm^2^/min)	2.48; 4.54 (1.03–14.18)	6.75; 3.67 (3.33–24.41)	63	< 0.01
DSA DAP (Gy*cm^2^)	67.6; 57.4 (16.7–292.9)	312.2; 75.1 (150.3–545.5)	78	< 0.01
DSA DAP/s (Gy*cm^2^/s)	1.02; 0.87 (0.22–4.61)	5.65; 2.88 (1.90–8.59)	82	< 0.01

Data represented as median and interquartile range (range)

### DF Image Quality

**Fig. 2:**
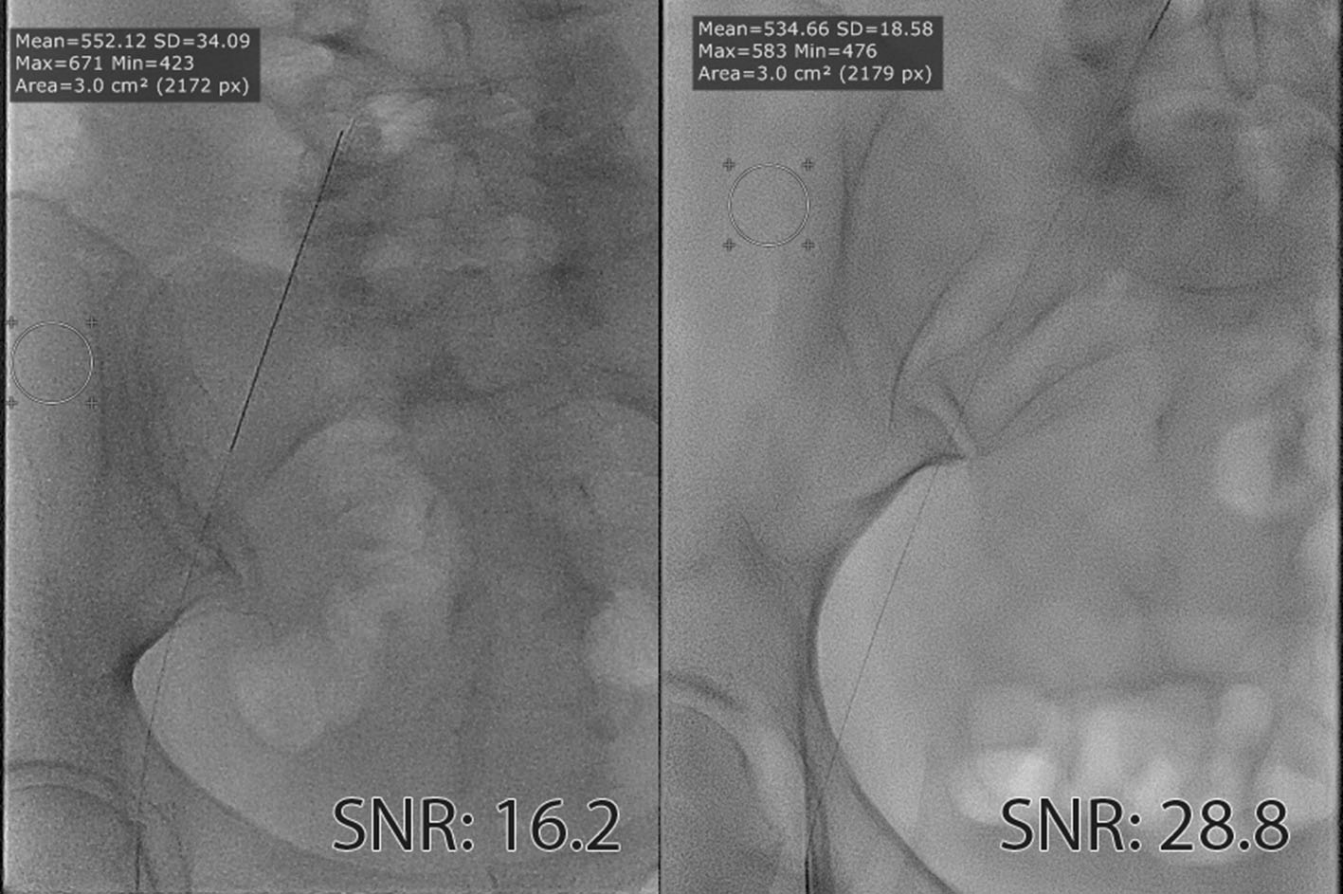
Digital fluoroscopy run of two patients acquired directly after access was gained via the common femoral artery. Left panel was acquired on the preceding imaging system and the right panel on the new imaging system. Both patients had similar body mass index and sagittal abdominal diameter. However, the old system had higher noise values (represented by a higher standard deviation) compared to the new system, resulting in a significantly lower signal-to-noise ratio of 16.2 versus 28.8

The SNR of the new system was significantly higher than that of the old system [30.8 ± 7.5 (range 14.7–44.4) vs. 23.5 ± 5.0 (range 14.8–31.2); *p* < 0.01] (Fig. 2). The visibility of the guidance wire expressed as CR on the other hand was similar in the new and the old system [1.78 ± 0.30 (range 1.34–2.56) vs. 1.92 ± 0.35 (range 1.19–2.49); *p* = 0.20].

### DSA Image Quality

**Fig. 3:**
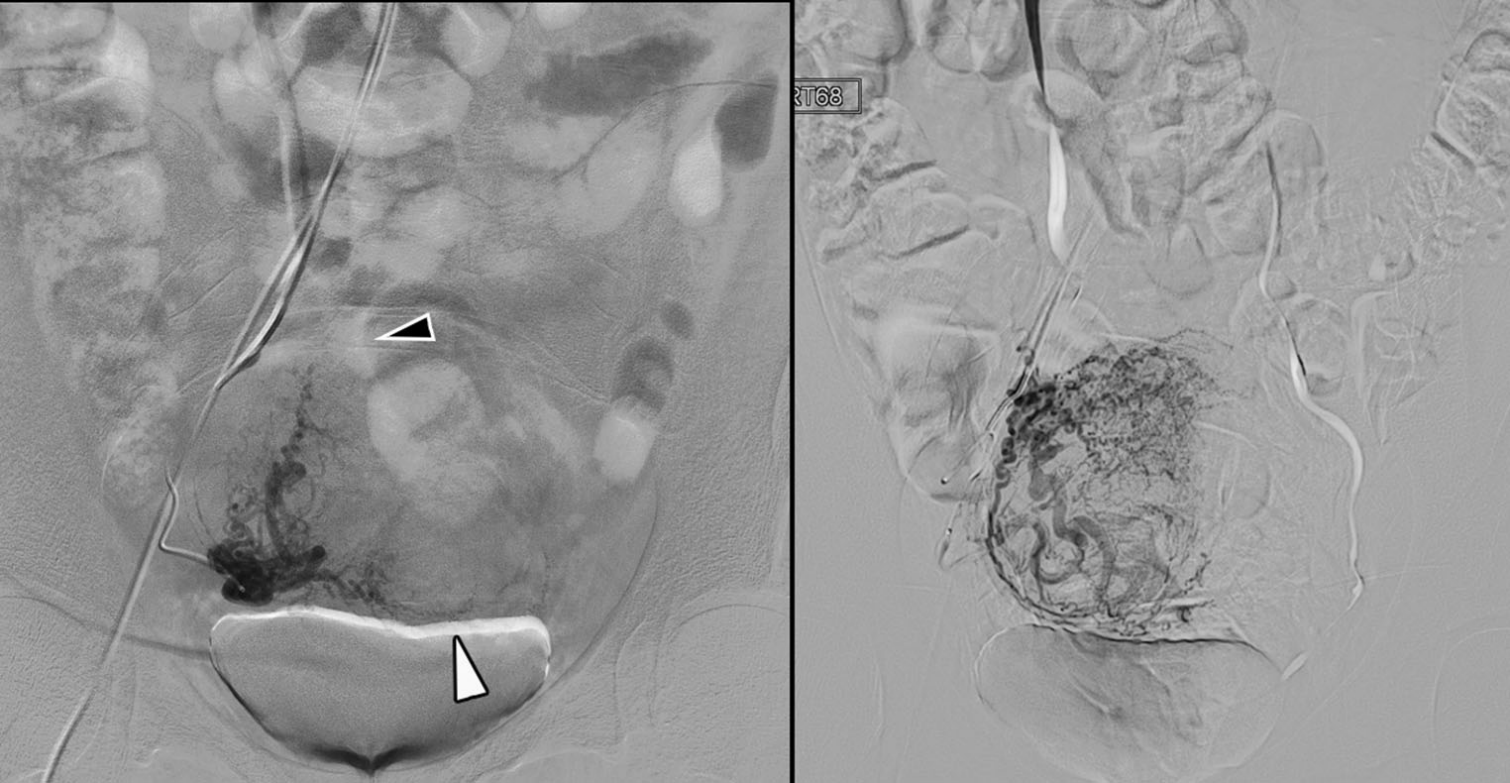
Digital subtraction angiography of the right uterine artery of two patients. Left panel was acquired on the preceding imaging system and the right panel on the new imaging system. Both patients had similar body mass index and sagittal abdominal diameter. The old system showed typical motion artifacts due to breathing (white arrowhead) and small bowel movement (black arrowhead). The automatic pixel shift algorithm of the new system compensated well both breathing and small bowel motion and facilitated the depiction of the small curling arteries within the uterine fibroids

According to both readers, the perfect depiction of the small feeding arteries was achieved more often by the new system (77.8 and 83.3%, respectively) than by the old system (both 55.6%). This difference was statistically significant for reader 2 (*p* = 0.03), but only a trend could be observed (*p* = 0.09) for reader 1. According to reader 1, the image quality was degraded by motion artifacts due to breathing and small bowel movement in 50% and in 83.3% on the old system, but only in 19.4 and 58.3% on the new system, respectively. Similarly, reader 2 observed motion artifacts due to breathing and small bowel movement in 66.7 and in 88.9% on the old system, but only in 13.9 and 61.1% on the new system, respectively. These differences were all statistically significant (*p* < 0.05), except for the small bowel motion artifacts observed by reader 1 (*p* = 0.07). In summary, the new system yielded a significantly better image quality than the old system according to reader 1 (median image quality score 2.0 vs. 1.5; *p* < 0.01) and reader 2 (median image quality score 2.0 vs. 1.0; *p* < 0.01) (Fig. 3). Kendall’s tau coefficient showed a strong agreement between both readers (correlation coefficient 0.67, *p* < 0.01). A detailed distribution of image quality scores is shown in Table 3.

**Table 3:** DSA image quality score cross-tables of both readers for the study and the control groups

		Reader 2				Total
		0	1	2	3	
Study group						
Reader 1	0	1	3	0	0	4
	1	0	1	3	0	4
	2	0	2	10	4	16
	3	0	0	5	7	12
Total		1	6	18	11	36
Control group						
Reader 1	0	6	0	0	0	6
	1	0	2	1	0	3
	2	0	4	4	0	8
	3	0	0	1	0	1
Total		6	6	6	0	18

## Discussion

The main finding of our study was the substantially improved image quality achieved by the real-time image processing of the new system. In particular, the amount of objectively measured image noise during DF runs was significantly lower for the new system (*p* < 0.01), whereas the visibility of the guidance wire was not affected (*p* = 0.20). The subjective image quality score of DSA runs was significantly higher for the new system (*p* < 0.01), corresponding to a better depiction of small tumor-feeding arteries and fewer motion artifacts due to breathing or small bowel movement. These results are in contrast to a recent study that reported similar image quality of the new and the preceding generation imaging system during UAE [15]. However, no objective DF image analysis by means of noise measurements was performed and DSA images with motion artifacts were excluded from subjective image evaluation in that study. In our opinion, motion artifacts are an important parameter of image quality and should be considered during image analysis. In theory, better image quality during DF could facilitate a faster catheterization of the target vessels. In addition, fewer motion artifacts in DSA runs could result in a lower amount of non-diagnostic DSA runs and reduce the necessity to repeat them. However, we did not observe any repetition of DSA runs and the DF radiation time was similar on both systems.

Another important finding was the significant radiation exposure reduction by two-thirds for the entire UAE procedure achieved by the new imaging system. Kohlbrenner et al. [15] recently reported a similar overall radiation exposure reduction during UAE with this new system. However, the authors reported only DAP and AK of the entire procedure and the impact of DF and DSA on the overall radiation exposure (reduction) was not evaluated.

Previous publications reported that DSA is the main contributor to overall radiation exposure during UAE [16], which was confirmed by the control cohort of our study, where DSA accounted for 62% of overall exposure. Thus, some colleagues suggested omitting aortography due to its low sensitivity to detect collateral supply from the ovarian arteries [17]. In the study group examined on the new imaging system, however, DSA had a lower impact on overall exposure than DF. Thus, aortography can be performed to identify collateral supply from different visceral arteries [18] with this new system without a significant increase in radiation exposure.

Our study had several limitations. First, the number of patients included in the control group was low (*n* = 18). However, even with a small sample size of 54 total patients, a statistically significant radiation exposure reduction was achieved, while the study and the control groups did not show any significant differences in patient characteristics and in radiation time. Second, RDSR was not available for the preceding imaging system; thus, DSA radiation time was calculated retrospectively using the number of images acquired during each DSA run. However, these calculations were verified by comparison with RDSR for a subset of patients examined on the new imaging system.

In conclusion, the new angiographic imaging system significantly improved image quality and reduced radiation exposure during UAE procedures.

## Electronic supplementary material

Below is the link to the electronic supplementary material.

Supplementary material 1 (WMV 2903 kb)

Supplementary material 2 (WMV 1254 kb)

Supplementary material 3 (DOCX 10 kb)
