# Supplementary material for: Characteristics of a New X-Ray Imaging System for Interventional Procedures: Improved Image Quality and Reduced Radiation Dose
Source: Cardiovasc Intervent Radiol. 2017 Oct 31;41(3):502–8. doi: 10.1007/s00270-017-1821-z (PMC5801377; doi:10.1007/s00270-017-1821-z)
Supplement: Supplementary file 3 — Supplementary material 3 (DOCX 10 kb) [file 270_2017_1821_MOESM3_ESM.docx]

Supplemental material. To highlight the differences in image quality between the old and the new system, video 1 & 2 show the entire DSA runs of the corresponding still images in figure 3, respectively.
